# Impact of bariatric surgery on ovarian reserve markers and its correlation with nutritional parameters and adipokines

**DOI:** 10.3389/fendo.2024.1284576

**Published:** 2024-03-15

**Authors:** Alba Andreu, Lilliam Flores, Marta Méndez, Ainize Ibarzabal, Gregori Casals, Imma Mercadé, Aina Borrás, Yasmina Barral, Inés Agustí, Dolors Manau, Josep Vidal, Gemma Casals

**Affiliations:** ^1^ Obesity Group, Endocrinology and Nutrition Department, Hospital Clinic de Barcelona, Barcelona, Spain; ^2^ Centro de Investigación Biomédica en Red Fisiopatología de la Obesidad y la Nutrición (CIBEROBN), Instituto de Salud Carlos III, Madrid, Spain; ^3^ Centro de Investigación Biomédica en Red de Diabetes y Enfermedades Metabólicas Asociadas (CIBERDEM), Instituto de Salud Carlos III, Madrid, Spain; ^4^ Fundació de Recerca Clínic Barcelona – Institut d’Investigacions Biomédiques August Pi i Sunyer (FRCB-IDIBAPS), Barcelona, Spain; ^5^ Human Assisted Reproduction Section, Hospital Clíınic de Barcelona, Universitat de Barcelona, Barcelona, Spain; ^6^ Gastrointestinal Surgery Department, Hospital Clınic de Barcelona, Barcelona, Spain; ^7^ Biomedical Diagnosis Center, Hospital Clínic de Barcelona, Barcelona, Spain

**Keywords:** fertility, reproduction, ovarian reserve, anti-Müllerian hormone, bariatric surgery, obesity

## Abstract

**Introduction:**

A reduction in anti-müllerian hormone (AMH) levels at short-term after bariatric surgery (BS) has been previously described. However, an assessment of ovarian reserve at longer-follow up, and a comprehensive evaluation of the potentially implicated factors has not been reported.

**Design:**

Prospective cohort study.

**Materials and methods:**

Twenty women aged 18-40 years with BMI 43.95 kg/m2 undergoing BS were studied at baseline (BS0), and at 1 month (BS1), 4 months (BS2), 12 months (BS3), and 24-36 months (BS4) after the surgery. Anthropometrics, reproductive hormones (AMH, FSH, LH, estradiol, testosterone, SHBG, androstenedione), metabolic parameters (adiponectin, leptin, ghrelin, insulin), and nutritional blood parameters (markers of nutritional status, vitamins, and minerals) were obtained at each study time point. Antral follicular count (AFC) was assessed by ultrasonography at BS0, BS3, and BS4. Mixed models were used for analysis of longitudinal data.

**Results:**

The mean AMH level was 3.88 ng/mL at BS0, decreased at BS3 (mean= 2.59 ng/mL; p=0.009), and remained stable between BS3 and BS4 (mean= 2.96 ng/mL; p=0.409). We also observed a non-significant decrease in AFC at BS3 (mean=26.14 at BS0, mean 16.81 at BS3; p=0.088) that remained stable at BS4 (mean= 17.86; p=0.731). Mixed models analysis showed: (a) a decrease in 10 kg of body weight was associated with an average decrease of 0.357 ng/mL in AMH (p=0.014); (b) a decrease in 1 BMI point was associated with an average decrease of 0.109 ng/mL in AMH (p=0.005); (c) an increase in 1 µg/mL of adiponectin was associated with an average decrease of 0.091 ng/ml in AMH (p=0.041) Significant positive correlations were found between the AMH levels after BS and plasma concentrations of testosterone, free androgen index, insulin and HOMA index. No significant correlations were detected between AMH levels and nutritional parameters.

**Conclusions:**

Our results were in line with previous observations, showing that AMH levels decreased significantly at 12 months after bariatric surgery, in parallel with a non-significant reduction in AFC. Both ovarian reserve markers showed a later stabilization up to the end of the study. Of note, postoperative AMH levels were positively correlated with key androgen and insulin resistance-related parameters.

## Introduction

The rising prevalence of obesity epidemic is associated with an increased risk of female subfertility (1, 2). In recent years, both animal model research and human clinical studies have shown that certain hormones produced by the digestive system (i.e. ghrelin) and the adipose tissue (i.e. leptin, adiponectin) would maintain a dialogue with reproductive hormones at different levels of the hypothalamic-pituitary-ovarian axis through a complex network of interactions (3). Among the reproductive hormones involved in this dialogue, anti-müllerian hormone (AMH) is crucial for ovarian folliculogenesis and has emerged as the most accurate hormonal marker of ovarian reserve, informing about the number of oocytes present within the ovaries of women at a specific moment of her life as well as the ovarian responsiveness to hormonal stimulation for *in vitro* fertilization (IVF) treatments (4, 5). Little is known about endogenous and exogenous factors potentially impacting serum AMH levels (6). AMH is significantly lower in women with obesity than in women with normal weight and is inversely correlated with BMI (7), although not all the studies reported differences between women with normal weight or obesity (8, 9). Different adipokines and nutrients may also influence its levels (10, 11). The main non-hormonal marker of ovarian reserve is the antral follicular count (AFC) assessed by gynecological ultrasound (12) and there is limited evidence on the impact of obesity on this marker (7).

Bariatric surgery (BS) is the most effective treatment for severe obesity. According to the latest international BS registry, over 77% of bariatric procedures are performed on women, half of whom are of reproductive age (13). Consequently, it is of paramount importance to improve our knowledge on the effects of BS on the determinants of fertility and reproduction. According to different investigations, AMH levels decrease significantly during the first months after BS (14–17) but currently, there is no available explanation for this reduction. It is also unknown whether the decrease in AMH reflects a reduction in the number of ovarian follicles implying a real loss of ovarian reserve or is a temporary functional alteration of the folliculogenesis that recovers after the period of maximum weight loss (WL). On the other hand, it has been shown that BS is associated with significant changes in plasmatic concentrations of adipokines and gut hormones, which also interact with the reproductive system (18). The aim of the present study was thus to investigate the potential modifications in plasma AMH levels and AFC, as the most accurate hormonal and non-hormonal ovarian reserve markers, up to 3 years after BS, and to identify potential factors involved in such modifications. To that aim, we performed a prospective study that included a comprehensive evaluation of reproductive hormones, adipokines, nutritional parameters and body composition, and we studied their potential associations with both AMH and AFC.

## Material and methods

### Study design

This prospective cohort study included 20 women aged 18–40 years old with obesity and indication of BS according to current ASMBS/IFSO criteria (19): BMI ≥ 40kg/m2 or ≥ 35 kg/m2 with associated major comorbidities. Patients were recruited from January 2016 to May 2017. The exclusion criteria were women with a previous hysterectomy or ovarian surgery, premature ovarian failure, pregnancy or active breastfeeding, treatment with hormonal contraceptives and/or other drugs with a known effect on ovulation or steroidogenesis in the previous 6 months (this restriction was to be maintained throughout the study). We used the Rotterdam criteria (20) for the polycystic ovarian syndrome (PCOS) definition, assuming the updated definition of PCOS morphology (21). All women were informed about the study protocol and a written informed consent was obtained from each participant. The study was approved by the Ethics Review Board of our center.

Patients were studied at 5 points: 1-3 months before surgery (baseline, BS0), 1 month after surgery (BS1), 4 months after surgery (BS2), 12 months after surgery (BS3) and between 24 - 36 months after surgery (BS4). The chronology of the study was based on the periods in which the main metabolic changes take place, including the period of rapid or maximum WL (12 months) and the subsequent stage of slower WL or weight stabilization (24-36 months) (22).

### Study parameters

Anthropometric assessment, body composition analysis, metabolic and hormonal analysis in plasma samples obtained in the fasting state, and gynecological ultrasound were performed at the per-protocol defined time points shown in **Table 1**. The points were scheduled between the 2nd and 4th day of the cycle in patients who maintained or recovered their menstrual cycles. In anovulatory patients, in which hormonal and ultrasonographic parameters were not affected by the phase of the menstrual cycle, their assessment coincided with the routine medical visits.

**Table 1:** Timeline´s protocol and parameters assessed at each time point.

		BS0	BS1	BS2	BS3	BS4
**Adipokines**	leptin,adiponectin, ghrelin, insulin, HOMA index.	X		X	X	
**Antrophometric assessment**	BW, BMI, WC, %TBWL, %EWL	X	X	X	X	X
**Bioimpedanciometry**	FFM, REE	X		X	X	
**Gynecological ultrasonography**	AFC	X			X	X
**Nutritional parameters**	VD3, Vitamin B12, ferritine, Prealbumin, CRP, total cholesterol	X		X	X	X
**Reproductive hormones**	AMH, FSH, LH, Testosterone, E2, FAI, SHBG, androstenedione	X	X	X	X	X

BW, body weight; BMI, body mass index; WC, waist circumference; %TBWL, total body weight loss; % EWL, excess weight loss; FFM, fat free mass; REE, resting energy expenditure; AMH, anti-Mullerian hormone; FSH, follicle-stimulating hormone; LH, luteinizing hormone; E2, 17 beta – estradiol, testosterone; FAI, free androgen index; SHBG, sex hormone binding globulin; VD3, 25-hydroxyvitamin D3; CRP, C- reactive protein; AFC, antral follicle count; BS0, baseline; BS1, 1 month after BS; BS2, 4 months after BS; BS3, 12 months after BS; BS4, 24-36 months after BS.

#### Anthropometric assessment

Body weight (BW) (kg), height (m) and waist circumference (WC) (cm) were recorded. Height and BW were measured using a calibrated scale without shoes and heavy outer garments to the nearest 0.5 cm and 0.1 kg, respectively. WC was measured at the midpoint between the last palpable lowest rib and the top of the iliac crest. BMI was calculated as BW divided by the square of height (kg/m^2^). Percentages of total body weight loss (TBWL) and excess weight loss (EWL) were calculated as follows: % TBWL = 100* (baseline BW – current BW)/baseline BW and % EWL = 100*(baseline BW – current BW)/baseline BW - ideal BW, the latter corresponding to the BW for a BMI of 25 kg/m^2^.

#### Bioelectrical impedance analysis

Body composition was assessed using bioelectrical impedance analysis (BIA, Tanita BC418), according to the manufacturer specifications and using the same impedentiometer: electrical impedance (ohms), fat free mass (FFM) (kg) and resting energy expenditure (REE) were obtained.

#### Biochemical analysis

Blood samples were collected after a 12-h overnight fast through antecubital vein puncture, processed immediately by centrifugation and stored at -80°C for subsequent analysis. All biochemical measurements were tested in the Biomedical Diagnosis Center of our center using an Atellica IM analyzer (Siemens Healthcare Diagnostics Inc., Tarrytown, USA). Nutritional parameters included 25-hydroxyvitamin D3 (VD3), vitamin B12, ferritin, prealbumin, C-reactive protein (CRP) and total cholesterol (Ct). Vitamin D sufficiency was considered when levels of VD3 were 30 ng/mL or more, insufficiency when VD3 levels ranged from 10 to 29 ng/mL, and deficiency was established when VD3 levels were less than 10 ng/mL (23). The deficiency values of the other nutritional parameters studied were based on normal ranges established by our center´s laboratory. Nutritional parameters were measured as previously reported (24, 25). The gut-hormone, adipokine, and metabolic assessment included the analysis of leptin, adiponectin, ghrelin and insulin levels and HOMA index in the fasting state. Leptin, adiponectin and ghrelin determinations were performed in serum using specific human ELISAs from DBC (Diagnostics Biochem Canada; London, Canada), Biovendor (Brno, Czech Republic) and Merck Millipore (Darmstadt, Germany), respectively. Insulin was determined by a two-site sandwich immunoassay using direct chemiluminescence technology (Atellica IM, Siemens Healthcare Diagnostics, Tarrytown, NY, USA), with an assay range of 0.5-300mU/L and intra- and inter-run CV of 3.8% and 4.6%, respectively. The analysis of reproductive hormones included AMH, follicle-stimulating hormone (FSH), luteinizing hormone (LH), 17 beta - estradiol (E2), testosterone, free androgen index (FAI), sex hormone binding globulin (SHBG) and androstenedione. AMH serum concentration was determined by chemiluminescence immunoassay with paramagnetic particles (ACCES2, Beckman Coulter, Brea, CA, USA). The detection limit was 0.02 ng/mL. The intra-series and inter-series CV were 1.6% and 3%, respectively. FSH and LH were determined by two-site sandwich immunoassays with direct chemiluminometric technology (Atellica IM, Siemens Healthcare Diagnostics, Tarrytown, NY, USA). For FSH, the determination range was 0.3-200 IU/L and the intra-series and inter-series CV were 2.4% and 1.5%, respectively; for LH, the determination range was 0.07-200 IU/L, and the intra-series and inter-series CV were 2.6% and 2.3%, respectively. Estradiol was determined by competitive electrochemiluminescence immunoassay (Atellica IM, Siemens Healthcare Diagnostics, Tarrytown, NY, USA), with a measurement range of 11.8-3000 pg/mL and intra- and inter-series CV <6%. Testosterone was analyzed by electrochemiluminescence immunoassay (ECLIA, Roche Diagnostics GmbH, Mannheim, Germany) with a measurement interval of 2.5-1500 ng/dL and intra-run CV of 2.8%. The free androgen index (FAI) was calculated according to the following equation: FAI = 100 × total testosterone (nmol/L)/SHBG (nmol/L). Androstenedione was measured by radioimmunoassay (DiaSource Diagnostics, Louvain-la-Neuve, Belgium) with a measurement interval of 3-1000 ng/dL and intra- and inter-series CV <6%.

#### Ultrasonographic study

The study of the AFC was performed by the same examiner using a Voluson S6 ultrasound machine (General Electric’s Medical Systems, Zipf, Austria), which incorporates a 5-7 MHz transvaginal transducer, and following the methodology described by Broekmans et al. (26). First, a conventional 2D scan of the pelvis was performed to exclude pathology and visualize the longitudinal and transverse planes of both ovaries. AFC was calculated as the sum of antral follicles in both ovaries. Antral follicles were defined as those measuring 2-10 mm in largest mean diameter on a 2-D plane.

### Statistical analyses

Descriptive statistics summarize characteristics for the overall sample at the next follow-up time points: baseline (BS0), 1 month (BS1), 4 months (BS2), 12 months (BS3) and 24-36 months after BS (BS4). Frequencies and percentages (%) were reported for categorical variables. Means and standard deviation (SD), as well as least square means (LSmeans) and standard error of means (SEM), were reported for continuous data. Repeated-measures ANOVA with pairwise comparisons were used for assessing differences in continuous variables, while McNemar’s non-parametric test was used for categorical variables. Mixed models were used for analysis of longitudinal data. The modelling included fixed effect terms for age at time of surgery, and clinical observations. A repeated measures term was included for the visits within each subject to consider multiple observations over time, expressed as continuous data. The comparative analysis between PCOS and non-PCOS patients was performed using the Wilcoxon test for paired data. A p <0.05 was considered statistically significant. All analyses were performed using Statistics Package for Social Sciences (SPSS) program version 22.0 (SPSS, IBM Corp. Released 2013, Armonk, NY) and R-Software program version 4.0.1 (27).

## Results

**Figure 1:**
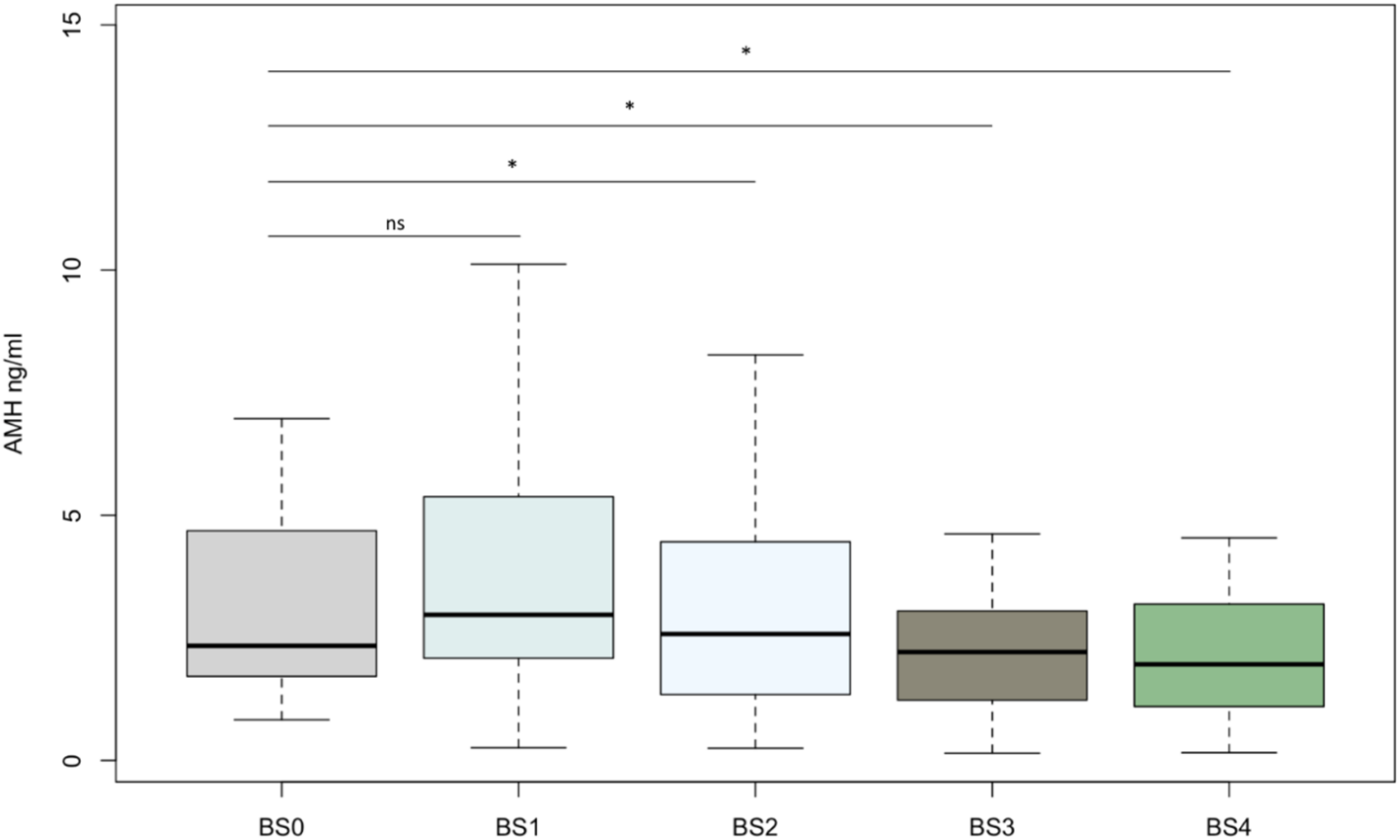
Boxplot showing anti-Mullerian hormone concentrations (ng/mL) for each time point. Each box represents the middle 50% of the data (25–75% range). The central horizontal line represents the median. Vertical lines represent the 10–90% range of data. Asterisks (*) among bars indicates a statistically significant difference between this point and the baseline status (p< 0.05). NS indicates non-significant differences (p>0.05). BS0, baseline; BS1, 1 month after BS; BS2, 4 months after BS; BS3, 12 months after BS; BS4, 24-36 months after BS; AMH, anti-Mullerian hormone.

**Figure 2:**
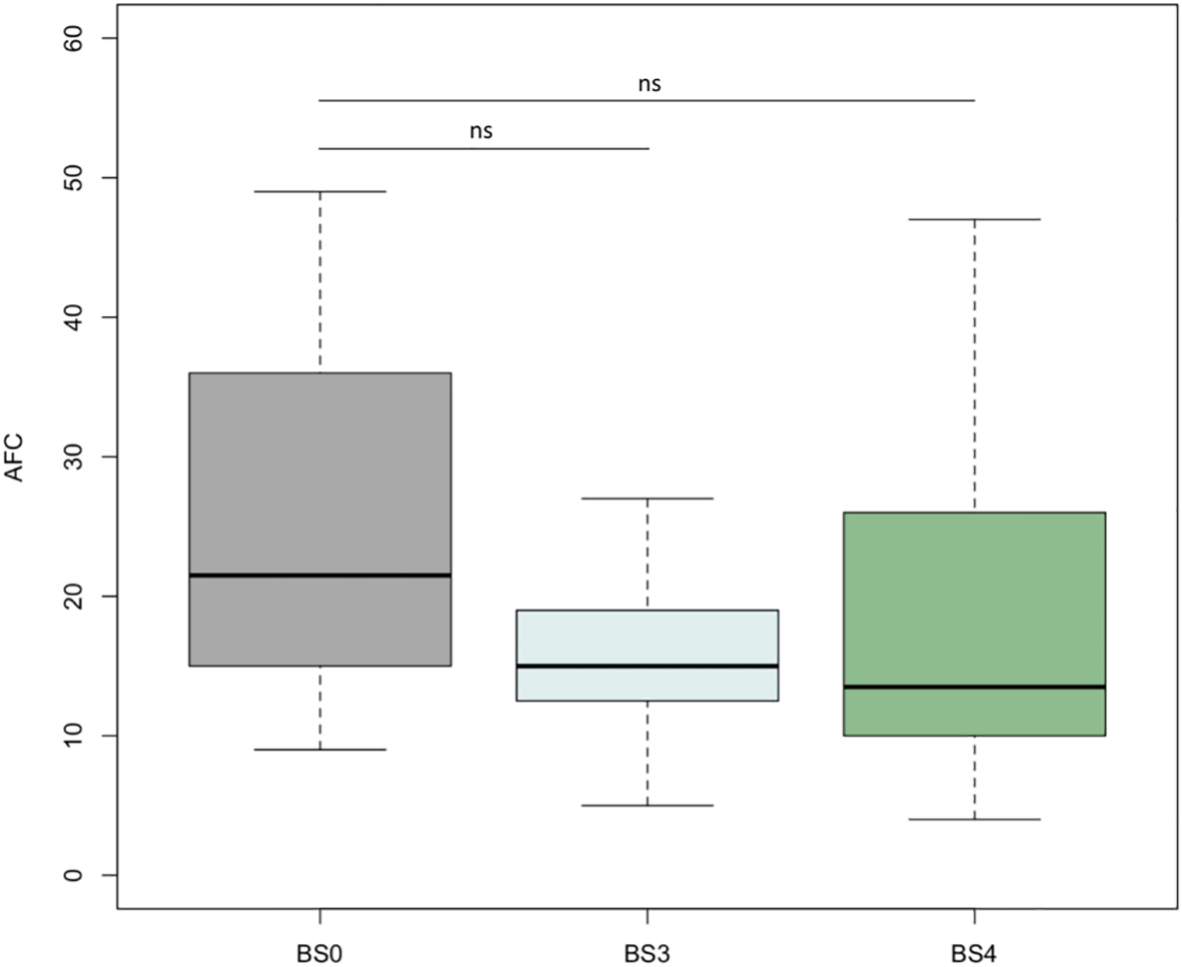
Boxplot showing antral follicle count for each time point. Each box represents the middle 50% of the data (25–75% range). The central horizontal line represents the median. Vertical lines represent the 10–90% range of data. NS indicates non-significant differences (p>0.05). BS0, baseline; BS3, 12 months after BS; BS4, 24-36 months after BS; AFC, antral follicle count.

The main baseline clinical characteristics of the patients included in the study are summarized in **Table 2**. The median age was 32.1 ± 4.4 years. Eight women had a history of infertility and 12 of them had PCOS. The mean BMI was 43.9 ± 5.1 kg/m^2^ at BS0 and 65% of them had class III obesity. Regarding the type of surgery, 14 patients underwent laparoscopic sleeve gastrectomy (LSG) and 6 underwent laparoscopic Roux-en-Y gastric bypass (LRYGB). The mean AMH level at BS0 was 3.88 ng/mL and, compared to BS0, decreased significantly at BS2 (mean= 2.63 ng/mL) (p=0.013), and at BS3 (mean=2.59 ng/mL) (p=0.009) (**Table 3**). There were no statistically significant differences in AMH levels between BS3 and BS4 (BS4 mean= 2.96 ng/mL). We observed a non-significant reduction in AFC from BS0 (mean= 26.14 ng/mL) to BS3 (mean= 16.81 ng/mL), with no further reduction beyond this point (BS4 mean= 17.86 ng/mL). The evolution of AMH levels and AFC during the period of study is represented in **Figures 1** and **2** respectively.

**Table 2:** Baseline clinical characteristics of the patients and type of surgery (n=20). Results in brakets are expressed as mean (SD) or proportion (%).

Parameter	
Age (years)	32.10 (4.41)
Infertility	8 (40%)
PCOS* (n %)	12 (60%)
Active smoker	1 (5%)
Previous-smoker	10 (50%)
BMI (kg/m2)	43.95 (5.08)
Obesity class	
II (BMI 35-40 kg/m2)	5 (25%)
III (BMI 40-50 kg/m2)	13 (65%)
IV (BMI > 50 kg/m2)	2 (10%)
WC (cm)	122.26 (11.72)
Type of surgery	
LSG	14 (70%)
LRYGB	6 (30%)

BMI, Body Mass Index; PCOS, Polycystic Ovarian Syndrome *According to Rotterdam criteria (20); WC, Waist Circumference; LSG, Laparoscopic Sleeve Gastrectomy; LRYGB, Laparoscopic Roux-en-Y-Gastric Bypass.

**Table 3:** Anthropometric and biochemical parameters during the first 12 months of the study and comparative analysis between different time points.

	BS0	BS1	BS2	BS3	BS0 vs BS1	BS0 vs BS2	BS0 vs BS3
**AMH** **(ng/mL)**	3.88 (0.69) [2.48; 5.27]	4.48 (0.69) [3.09; 5.87]	2.63 (0.71) [1.20; 4.06]	2.59 (0.7) [1.17; 4.01]	0.192	**0.013**	**0.009**
**BW** **(kg)**	117.52 (3.34) [110.82-124.23]	104.54 (3.34) [97.84;111.24]	92.10(3.34) [85.40-98.80]	79.30 (3.34) [72.60; 86.00]	**<0.01**	**<0.01**	**<0.01**
**EWL** **(%)**		27.2 (3.44) [20.3; 34.1]	53.72 (3.44) [46.82; 60.62]	79.98 (3.44) [73.08; 86.88]	**<0.01**	**<0.01**	**<0.01**
**TBWL** **(%)**		11.18 (3.92) [9.27;12.95]	22.08(4.59) [19.93;24.24]	32.86 (6.48) [29.83;35.90]	**<0.01**	**<0.01**	**<0.01**
**WC** **(cm)**	122.26 (11.72) [116.61;127.91]		101.47(2.99) [95.20;107.75]	93.81(12.21) [86.42;101.19]		**<0.001**	**<0.001**
**FFM** **(kg)**	57.85 (1.04) [55.45; 60.26]		52.67 (1.11) [50.12; 55.22]	50.62 (1.14) [47.99; 53.26]		**<0.01**	**<0.01**
**BMI** **(kg/m2)**	43.85 (1.14) [41.57; 46.13]	38.98 (1.14) [36.70;41.25]	34.32 (1.14) [32.04; 36.60]	29.59 (1.14) [27.31;31.86]	**<0.01**	**<0.01**	**<0.01**
**Adiponectin (mcg/mL)**	7.31 (1.24) [4.83; 9.8]	8.46 (1.24) [5.97;10.94]	10.51 (1.31) [7.87; 13.14]	15.31 (1.28) [12.73; 17.89]	0.371	**0.022**	**<0.01**
**Leptin (ng/mL)**	60.15 (5.52) [49.04; 71.26]	35.35 (5.52) [24.24; 46.46]	24.46 (5.95) [12.49; 36.44]	21.5 (5.80) [9.83; 33.16]	**<0.01**	**<0.01**	**<0.01**
**Ghrelin** **(pg/mL)**	998.73 (72.64) [852.60; 1144.86]	735.20 (72.64) [589.07; 881.33]	820.42 (77.06) [666.40; 975.45]	862.41 (75.47) [710.98; 1014.63]	**<0.01**	**0.030**	0.087
**Insulin** **(mU/L)**	46.42 (7.33) [30.81; 62.04]			10.55 (7.95) [6.89; 26.99]			**0.004**
**HOMA**	11.93 (2.12) [7.40; 16.45]	-	-	1.97 (2.30) [2.93; 6.88]			**0.006**
**Testosterone**	33.13 (3.03) [26.66; 39.59]			28.03 (3.15) [21.31; 34.74]			**0.046**
**FAI**	4.38 (0.45) [3.42; 5.34]			1.68 (0.49) [0.64; 2.71]			**<0.01**
**SHBG** **(nmol/L)**	34.11 (7.07) [19.05; 49.17]			72.72 (7.57) [56.57; 88.086]			**<0.001**
**AFC**	26.14 (3.64) [18.28;34.01]	-	-	16.81 (2.03) [11.42; 26.32]			0.088

Results expressed as LSMeans (SEM) [95%CI].

BW, body weight; % EWL, excess weight loss; %TBWL, total body weight loss; WC, waist circumference; FFM, fat free mass; BMI, body mass index; AMH, anti-Mullerian hormone; FAI, free androgen index; SHBG, sex hormone binding globulin; AFC, antral follicle count.

BS0, baseline; BS1, 1 month after BS; BS2, 4 months after BS; BS3, 12 months after BS.

Bold values indicate a statistically significant difference between different time points (p< 0.05).

The results of the main anthropometric and biochemical parameters in the first 12 months of the study are summarized in **Table 3**. We detected a significant decrease of leptin levels (BS0 mean= 60.15 ng/mL, BS3 mean=21.5 ng/mL; p <0.01) and a significant increase in the adiponectin levels (BS0 mean=7.31mcg/mL, BS3 mean=15.31 mcg/mL; p <0.01). We also observed a significant reduction between BS0 and BS3 in insulin levels (BS0 mean=46.42 mU/L, BS3 mean=10.55 mU/L; p=0.004), HOMA index (BS0 mean=11.93, BS3 mean=1.97; p=0.006), testosterone levels (BS0 mean=33.13 ng/dL, BS3 mean=28.03 ng/dL; p=0.046) and FAI (BS0 mean=4.38, BS3mean=1.68; p <0.01), and a significant increase in SHBG levels (BS0 mean= 34.11 mmol/L, BS3 mean= 72.72 mmol/L; p <0.001). We also analyzed the data of women with PCOS and without PCOS separately, observing significant changes in the postoperative levels of AMH, leptin and adiponectin only in the PCOS group, while a significant decrease of FAI was observed in both groups (**Supplementary Table 1**).

The main nutritional parameters and their deficiencies in the first 12 months of the study are summarized in **Table 4**. We observed a significant increase in VD3 and vitamin B12 levels and a significant decrease in CRP concentrations from baseline to BS3.

**Table 4:** Nutritional parameters and their deficiencies at baseline and 12 months after BS and comparative analysis between these time points.

	BS0	BS3	BS0 vs BS3
**Vitamin D3 (ng/mL)**	17.84 (6.86)	31.62 (16.18)	**0.004**
**VD3 < 30 ng/mL [n,(%)]**	19 (95%)	13 (65%)	**0.034**
**Vitamin B12 (pg/mL)**	418.55 (133.28)	656.55 (351.66)	**0.003**
**VB12 <299 pg/mL [n,(%)]**	4 (20%)	0 (0%)	**0.046**
**Ferritin (ng/mL)**	71.20 (66.76)	93.89 (70.30)	0.122
**Ferritin <15 ng/mL [n,(%)]**	4 (20%)	5 (25%)	0.157
**Prealbumin (g/L)**	0.23 (0.03)	0.21 (0.04)	0.103
**Prealb < 0.200 g/L [n,(%)]**	3 (15%)	9 (45%)	0.058
**CRP (mg/dL)**	1.19 (0.85)	0.27 (0.37)	**<0.001**
**CRP ≥ 0.5 mg/dL [n,(%)]**	16 (80%)	3 (15%)	**<0.001**
**Ct(mg/dL)**	171.80 (28.00)	163.40 (28.72)	0.259
**Ct ≥ 200 mg/dL) [n,(%)]**	3 (15%)	4 (20%)	0.655

Results expressed as LSMeans (SEM).

VD3, 25-hydroxyvitamin D3; VB12, Vitamin B12; Prealb, Prealbumin; CRP, C-reactive protein; Ct, total cholesterol.

BS0, baseline; BS3, 12 months after BS.

Bold values indicate a statistically significant difference between different time points (p< 0.05).

**Figure 3:**
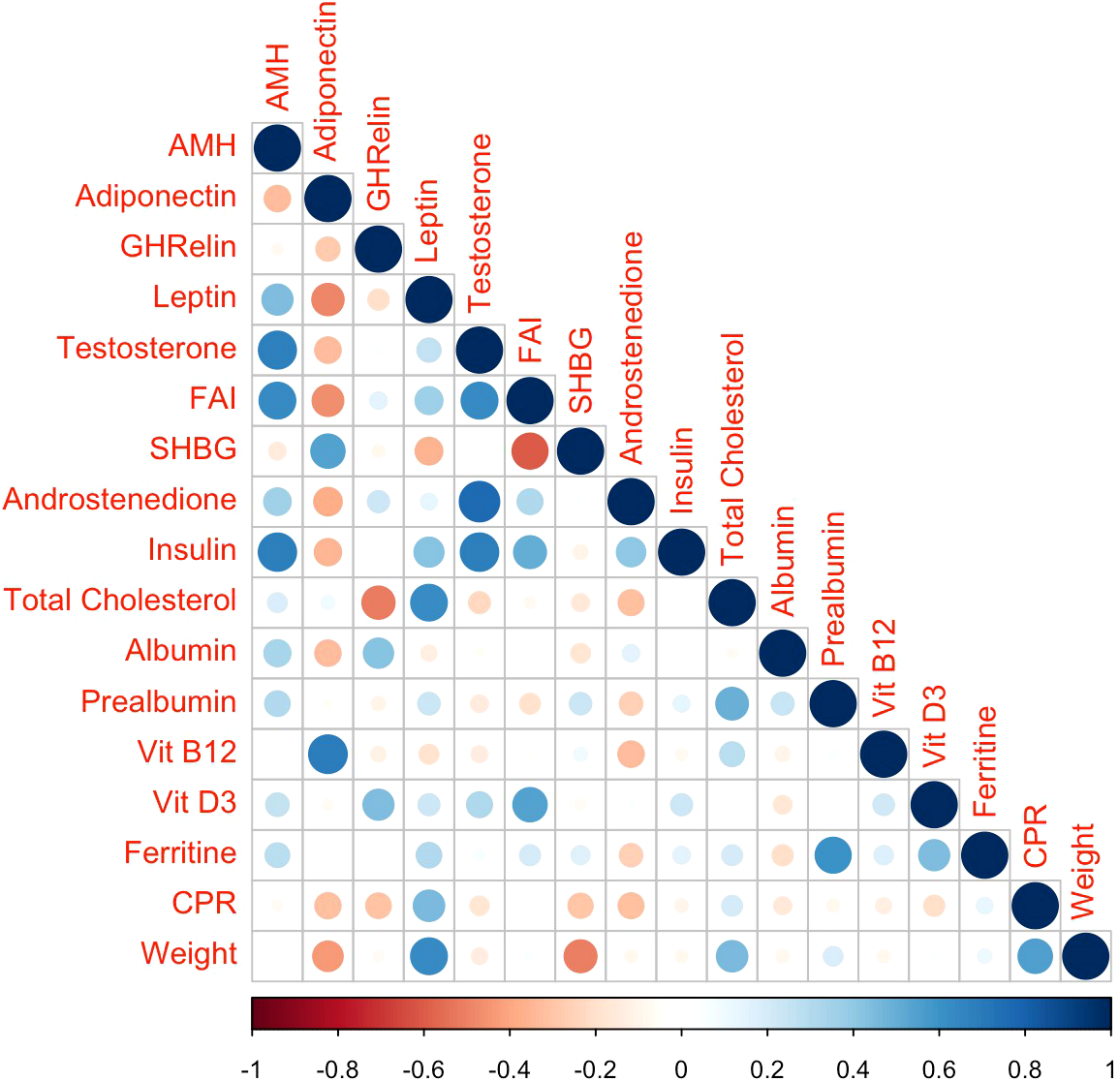
Correlation plot for the associations between biochemical variables at 12 months after BS. The symmetric correlation matrix was created using the R “corrplot” package. The colors represent the degree of pairwise correlation according Pearson’s correlation coefficient. AMH, anti-Mullerian hormone; FAI, free androgen index; SHBG, sex hormone binding globulin; Vit D3, 25-hydroxyvitamin D3; CPR, C-reactive protein.

The statistical correlations between AMH levels at BS3 or its modification from BS0 to BS3 and the main reproductive and metabolic parameters are shown in **Figure 3** and **Supplementary Table 2**. At BS3, we observed significant correlations between AMH and androgen levels (testosterone: R= 0.676, p= 0.003; FAI: R= 0.631, p = 0.007), insulin levels (R= 0.684, p=0.002) and HOMA index (R= 0.736, p= 0.001). We also found a significant correlation between the percentage of change in AMH and androstenedione concentrations (R= 0.632, p= 0.009), as well as BIA data (REE: R= 0.973, p= 0.027; FFM: R= 0.984, p=0.016). No significant correlations were detected between AMH levels and any nutritional parameter or adipokine at any study time point.

A longitudinal analysis using mixed models revealed the following associations: a) a decrease in 10 kg of BW was associated with an average decrease of 0.357 ng/mL in AMH p= 0.014); (b) a decrease in 1 BMI point was associated with an average decrease of 0.109 ng/mL in AMH (p=0.005); (c) an increase in 1 µg/ml of adiponectin was associated with an average decrease of 0.091 ng/mL in AMH (p= 0.041).

## Discussion

The present investigation studied the evolution of hormonal and non-hormonal ovarian reserve markers, as well as different reproductive, nutritional, anthropometric, and metabolic factors after BS, including the period of rapid or maximum WL until the period of slower WL or weight stabilization. To our knowledge, this is the longest postoperative follow-up after BS including the study of ovarian reserve markers. Our results confirmed a significant reduction in AMH levels 12 months after BS and demonstrate, for the first time, its subsequent stabilization. Furthermore, we also observed a parallel, albeit non-statistically significant, reduction and subsequent stabilization in AFC. Significant correlations were found between AMH levels as well as its changes after BS, and different reproductive and metabolic factors.

The main objective of our investigation was to analyze the evolution of AMH serum levels and the ultrasonographic AFC as the main ovarian reserve markers from surgery to the period of weight stabilization. AMH is produced by granulosa cells of small, growing follicles in the ovary and acts as a follicular gatekeeper, regulating the transition of primordial follicles into the growing follicle pool, becoming a regulator in follicle development in the transition process to dominance (4). Serum AMH levels strongly correlate with the number of growing follicles, being considered the most accurate hormonal ovarian reserve marker, predicting ovarian stimulation response for *in vitro* fertilization cycles, and helping to determine the dose of gonadotropins for these treatments (6). Serum AMH concentration assesses the “functional ovarian reserve,” a term that is preferred over “ovarian reserve,” since it reflects the pool of growing follicles that can potentially ovulate (6).

The significant reduction of AMH serum levels observed during the WL phase after BS observed in our study is in line with that reported by other authors (15–18). Admittedly, other studies have shown contradictory results detecting a significant increase of AMH levels after BS (28–30). However, the period of evaluation in these studies was restricted to the first 6 months after BS, and these discrepant results may also be related to methodological differences among studies. Indeed, we detected an initial increase in AMH levels in the first postoperative 30 days. We did not analyze the parameters at 6 months but at the postoperative 4^th^ month we already observed a significant decrease in line with that observed by other authors (14, 16, 17). Importantly, in our study we measured AMH plasma concentrations beyond the rapid WL phase after BS. Thus, our data adds to the field the description of AMH serum concentrations up to 3 years after BS. As a robust marker of ovarian reserve, the observed decrease in AMH concentrations after BS could suggest a reduction in the number of ovarian follicles. Nonetheless, AMH levels must be interpreted with caution and in the context of the endocrine environment. Thus, it cannot be ruled out that AMH may not truly reflect ovarian reserve after BS, at time when major endocrine, metabolic, anthropometric and nutritional changes occur.

In women with obesity, adipose tissue excess and dysfunction may contribute to androgen excess through insulin resistance and, possibly, by proinflammatory cytokines secreted into the circulation (31). In women with obesity and PCOS, serum androgen concentrations normalize after BS, with improvement and even resolution of signs such as hirsutism and symptoms of menstrual dysfunction. The high resolution rate of PCOS, above 95%, achieved with WL further supports the causal role of obesity and adipose tissue dysfunction in gonadal failure characteristics of women with obesity (31). Different endocrine or metabolic contexts may influence AMH serum levels. Plasmatic and intrafollicular levels of AMH are significantly increased in PCOS women (32) and, according to the most recent international guidelines, AMH can be used for the diagnosis of PCOS instead of ultrasound (33). Moreover, there is growing evidence that AMH may play a pathophysiological role in PCOS (34, 35). Regarding the influence of weight, AMH is significantly lower in women with obesity than in women with normal weight and is inversely correlated with BMI (36), although more recent meta-analyses show conflicting data (8, 9). Moreover, there are conflicting results regarding the impact of WL strategies on AMH levels, either with lifestyle interventions (37–40) or with pharmacological treatment (41, 42). According to data from a RCT exploring the serum AMH levels in response to diet and/or physical exercise in women with BMI ≥25 kg/m2 and PCOS, AMH would decrease following a dietary but not an exercise-based intervention (37). In this study, performed in women with PCOS, the reduction in AMH seemed not to be explained by a decrease in the number of ovarian follicles, but rather to a decreased production of AMH by each follicle (37). Likewise, reduced AMH levels have been reported following a very low-calorie ketogenic diet (38). On the contrary, no change in AMH was found following dietary interventions aiming at WL in other studies (39, 40), with these results being independent of the PCOS status (40). Contradictory results in AMH levels have also been reported following lifestyle interventions combined either with sibutramine (41) or orlistat (42) in women with obesity.

Finally, whether the effects of BS on AMH differ from those of other WL interventions is an unresolved question. Indeed, BS induces large ponderal changes coupled with profound metabolic modifications, which may explain the postoperative reduction of the AMH (43). The AMH stability beyond the first 12 months after BS could be explained because the main determinant of the decrease in AMH levels in our study was WL, which tended to be more stable after the initial period of sharp decrease.

To overcome this limitation, we included a sub-analysis of the data by separating women with PCOS from those without PCOS (**Supplementary Table 1**). Contrary to the literature stating that AMH levels are inversed correlated to BMI (7, 36), in our study women with obesity had high AMH levels before BS, regardless of PCOS status. Moreover, they decreased after BS in both PCOS and non-PCOS subgroups, suggesting that high AMH levels are probably a common feature of women with obesity, possibly related to metabolic or hormonal alterations specific to the obesity condition. Our findings reveal a significant postoperative decrease of AMH, insulin and androgen levels and an increase in adiponectin. All these hormones are involved in the regulation of the follicular development, which is impaired in women with obesity and especially in the context of PCOS (32, 44, 45). In these patients, WL may improve the follicular development, the ovulatory function and the reproductive outcomes by means of the associated reduction of AMH, insulin resistance and androgen excess and the increase of adiponectin along with their effects at both the reproductive hormone and follicular levels. In our study, at 12 months after BS, we detected a significant positive correlation between AMH and androgen levels on the one hand and insulin sensitivity on the other. Additionally, AMH decrease from BS0 to BS3 was significantly positively correlated with androstenedione levels and body composition data (FFM and REE). A decline in testosterone levels has been involved in the reduction of AMH following dietary or pharmacological interventions (37, 41, 42). The potential role of testosterone in this effect is suggested by studies showing that pharmacologically induced increase in intrafollicular androgen levels by letrozole is associated with increased granulosa cell production of AMH (46). Other authors pointed out that the observed postoperative decline in AMH and testosterone levels might be related to the improved insulin sensitivity that occurs after BS (16). The postoperative reversion of insulin resistance may impact granulosa cells function and consequently alter AMH concentration (10). According to the results of an animal model study, insulin could enhance follicular recruitment and promote follicular development in synergy with FSH once follicles acquire FSH sensitivity (47). However, conflicting results have been reported regarding the association between AMH and surrogate markers of insulin resistance, such as the HOMA index (37). It is also possible that adiponectin and leptin were involved in modulating AMH levels and/or ovarian function, as these adipokines play a role in reproductive processes acting on the hypothalamic-pituitary-ovarian axis (10). Indeed, there are adiponectin and leptin receptors in the granulosa cells of the ovaries (10). We found an inverse association between changes in adiponectin and AMH levels during the first postoperative 12 months but, in accordance with a previous study (17), we did not detect a significant correlation between AMH levels or postoperative modifications and leptin or adiponectin serum concentrations. Although ghrelin may also influence AMH levels (48), we did not detect an association between these two parameters. Finally, regarding the potential influence of postoperative nutritional deficiencies with AMH synthesis, the most investigated parameter is vitamin D. A metanalysis revealed discrepant findings in cross-sectional studies regarding an association between serum vitamin D and AMH levels (11). In particular, according to this metanalysis, the relationship between vitamin D supplementation and serum AMH concentrations seems to depend on the ovulatory status of the patient: while AMH levels significatively increased following vitamin D supplementation in ovulatory women without PCOS, they significantly decreased following supplementation in PCOS women, maybe reflecting the ability of vitamin D to improve folliculogenesis in PCOS. However, a real impact on the ovarian reserve cannot be discarded (11). In our study, no significant correlation was detected between AMH levels and any nutritional parameter, suggesting that nutritional care following BS is unlike to influence the observed reduction in ovarian reserve markers, in accordance with another investigation (17).

To overcome the potential limitations of AMH as marker of ovarian reserve in such a context of important metabolic changes, as described above, in our study we also measured AFC as a reliable non-hormonal marker of ovarian reserve (12). While AMH is produced by granulosa cells of primary and secondary follicles and mostly by small antral follicles, AFC pertains only to large preantral and small antral follicles (49). Comparisons of AFC and AMH levels have generally yielded a similar good predictive value for ovarian stimulation response (50). In agreement with the changes in AMH at 12 months after surgery, we also detected a reduction, albeit non-significant, of AFC from baseline to 12 months after BS. Discrepancy in the statistical significance in the changes of AMH and AFC throughout the study period may be related to the greater intracycle and inter-cycle variation of the AFC in women with obesity (51) or to the limited sample size.

The main strengths of the present study are the assessment of ovarian reserve using two different biomarkers, as well as a comprehensive analysis of several reproductive, anthropometric, nutritional, and metabolic factors potentially associated with the modification of AMH serum concentrations after BS. Furthermore, previous investigations on AMH levels after BS were restricted to the first postoperative 6-12 months, that is the period in which the major anthropometric and metabolic changes take place. Thus, another strength of our study is that the follow-up of our participants was extended until the period of slower WL or weight stabilization, beyond the period of these profound changes. Nonetheless, our study is not without limitations. We acknowledge that our sample size is small, but it would be difficult to perform such an exhaustive study with a larger sample, including so many variables. The lack of a control group of women with similar age and baseline BMI who did not undergo BS is another limitation. However, it would have been difficult to recruit women with these characteristics who would agree not to undergo surgery for such a long period of time. Another limitation of the present study is the high percentage of patients with a diagnosis of PCOS, which could be a potential confounding factor. Contrary to the literature stating that AMH levels are inversed correlated to BMI (7, 36), in our study women with obesity had high AMH levels before BS, regardless of PCOS status. Moreover, they decreased after BS in both PCOS and non-PCOS subgroups, suggesting that high AMH levels are probably a common feature of women with obesity, possibly related to metabolic or hormonal alterations specific to the obesity condition. Although the modifications in AMH and adipokines levels were significant only in the PCOS subgroup, the number of patients in each group was probably too small to draw solid conclusions. It could also be argued that the limited accuracy of the analysis of body composition using BIA may have limited our ability to assess its relationship with functional ovarian reserve markers (52). Finally, since our findings are based on associations, causality for the post-surgical changes in ovarian reserve could not be established.

## Conclusions

We confirmed a significant decrease in AMH levels and a downward trend in AFC during the first 12 months after BS, followed by a subsequent stabilization of both ovarian reserve markers. AMH variations were associated with different reproductive and metabolic parameters, especially with androgen and insulin modifications. It remains unclear whether these changes were due to an impact on follicular physiology and granulosa cell function or whether they corresponded to an effective quantitative loss of ovarian follicles. From a clinical perspective, it is important to underline that ovarian reserve markers inform about the number of oocytes present in the ovaries of women at a specific moment of their life and the capacity of ovarian response to IVF treatments: AMH is a marker of quantity, but not quality. Thus, if the AMH reduction observed after BS corresponded to a real reduction of ovarian reserve, this might imply a lower response to ovarian stimulation in assisted reproduction techniques, but it would not necessarily be associated with a further reduced reproductive capacity or risk of future infertility.

## Data availability statement

The original contributions presented in the study are included in the article/**Supplementary Material**. Further inquiries can be directed to the corresponding authors.

## Ethics statement

The studies involving humans were approved by ethic research committee at Hospital Clinic of Barcelona (HCB/2023/0134). The studies were conducted in accordance with the local legislation and institutional requirements. The participants provided their written informed consent to participate in this study.

## Author contributions

AA: Conceptualization, Data curation, Investigation, Methodology, Project administration, Resources, Writing – original draft, Writing – review & editing. LF: Data curation, Funding acquisition, Investigation, Validation, Writing – review & editing. MM: Data curation, Formal Analysis, Investigation, Methodology, Project administration, Writing – review & editing. AI: Data curation, Investigation, Writing – review & editing. GrC: Data curation, Investigation, Validation, Writing – review & editing. IM: Data curation, Investigation, Validation, Writing – review & editing. AB: Data curation, Investigation, Validation, Writing – review & editing. YB: Data curation, Investigation, Validation, Writing – review & editing. IA: Data curation, Investigation, Validation, Writing – review & editing. DM: Data curation, Validation, Writing – review & editing. JV: Conceptualization, Data curation, Methodology, Project administration, Supervision, Writing – review & editing. GeC: Conceptualization, Data curation, Funding acquisition, Investigation, Methodology, Project administration, Supervision, Writing – original draft, Writing – review & editing.
