# Supplementary material for: Impact of bariatric surgery on ovarian reserve markers and its correlation with nutritional parameters and adipokines
Source: Front Endocrinol (Lausanne). 2024 Mar 15;15:1284576. doi: 10.3389/fendo.2024.1284576 (PMC10978777; doi:10.3389/fendo.2024.1284576)
Supplement: Supplementary file 1 [file Presentation_1.pptx]

## Slide 1
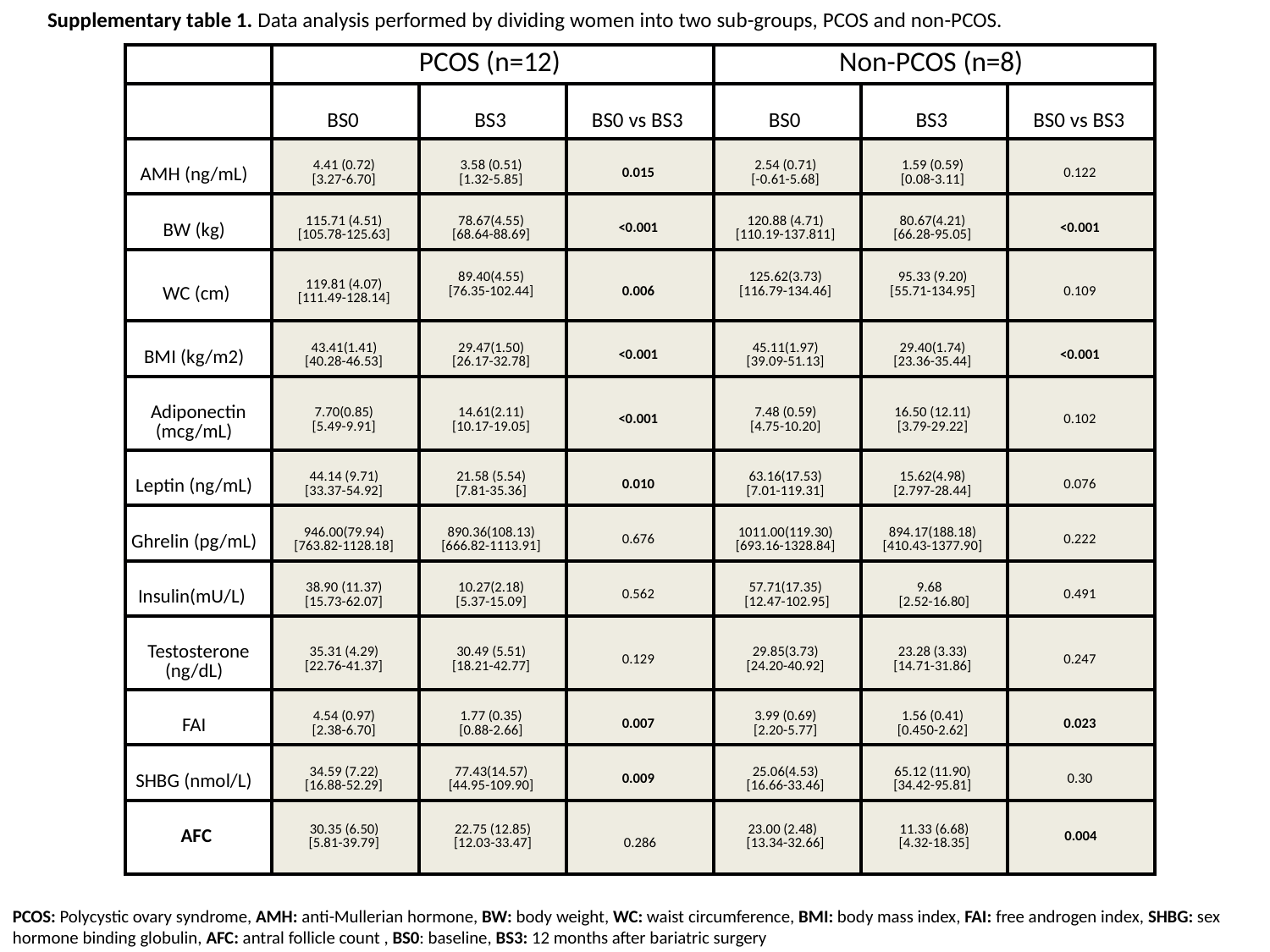

Supplementary table 1. Data analysis performed by dividing women into two sub-groups, PCOS and non-PCOS.
| | PCOS (n=12) | | | Non-PCOS (n=8) | | |
| --- | --- | --- | --- | --- | --- | --- |
| | BS0 | BS3 | BS0 vs BS3 | BS0 | BS3 | BS0 vs BS3 |
| AMH (ng/mL) | 4.41 (0.72)  [3.27-6.70] | 3.58 (0.51)  [1.32-5.85] | 0.015 | 2.54 (0.71)  [-0.61-5.68] | 1.59 (0.59)  [0.08-3.11] | 0.122 |
| BW (kg) | 115.71 (4.51)  [105.78-125.63] | 78.67(4.55)  [68.64-88.69] | <0.001 | 120.88 (4.71)  [110.19-137.811] | 80.67(4.21)  [66.28-95.05] | <0.001 |
| WC (cm) | 119.81 (4.07)  [111.49-128.14] | 89.40(4.55)  [76.35-102.44] | 0.006 | 125.62(3.73)  [116.79-134.46] | 95.33 (9.20)  [55.71-134.95] | 0.109 |
| BMI (kg/m2) | 43.41(1.41)  [40.28-46.53] | 29.47(1.50)  [26.17-32.78] | <0.001 | 45.11(1.97)  [39.09-51.13] | 29.40(1.74)  [23.36-35.44] | <0.001 |
| Adiponectin (mcg/mL) | 7.70(0.85)  [5.49-9.91] | 14.61(2.11)  [10.17-19.05] | <0.001 | 7.48 (0.59)  [4.75-10.20] | 16.50 (12.11)  [3.79-29.22] | 0.102 |
| Leptin (ng/mL) | 44.14 (9.71)  [33.37-54.92] | 21.58 (5.54)  [7.81-35.36] | 0.010 | 63.16(17.53)  [7.01-119.31] | 15.62(4.98)  [2.797-28.44] | 0.076 |
| Ghrelin (pg/mL) | 946.00(79.94)  [763.82-1128.18] | 890.36(108.13)  [666.82-1113.91] | 0.676 | 1011.00(119.30)  [693.16-1328.84] | 894.17(188.18)  [410.43-1377.90] | 0.222 |
| Insulin(mU/L) | 38.90 (11.37)  [15.73-62.07] | 10.27(2.18)  [5.37-15.09] | 0.562 | 57.71(17.35)   [12.47-102.95] | 9.68     [2.52-16.80] | 0.491 |
| Testosterone (ng/dL) | 35.31 (4.29)  [22.76-41.37] | 30.49 (5.51)  [18.21-42.77] | 0.129 | 29.85(3.73)  [24.20-40.92] | 23.28 (3.33)  [14.71-31.86] | 0.247 |
| FAI | 4.54 (0.97)  [2.38-6.70] | 1.77 (0.35)  [0.88-2.66] | 0.007 | 3.99 (0.69)  [2.20-5.77] | 1.56 (0.41)  [0.450-2.62] | 0.023 |
| SHBG (nmol/L) | 34.59 (7.22)  [16.88-52.29] | 77.43(14.57)  [44.95-109.90] | 0.009 | 25.06(4.53)  [16.66-33.46] | 65.12 (11.90)  [34.42-95.81] | 0.30 |
| AFC | 30.35 (6.50)  [5.81-39.79] | 22.75 (12.85) [12.03-33.47] | 0.286 | 23.00 (2.48)  [13.34-32.66] | 11.33 (6.68) [4.32-18.35] | 0.004 |
PCOS: Polycystic ovary syndrome, AMH: anti-Mullerian hormone, BW: body weight, WC: waist circumference, BMI: body mass index, FAI: free androgen index, SHBG: sex hormone binding globulin, AFC: antral follicle count , BS0: baseline, BS3: 12 months after bariatric surgery
