# Supplementary material for: Impact of bariatric surgery on ovarian reserve markers and its correlation with nutritional parameters and adipokines
Source: Front Endocrinol (Lausanne). 2024 Mar 15;15:1284576. doi: 10.3389/fendo.2024.1284576 (PMC10978777; doi:10.3389/fendo.2024.1284576)
Supplement: Supplementary file 2 [file Presentation_2.pptx]

## Slide 1
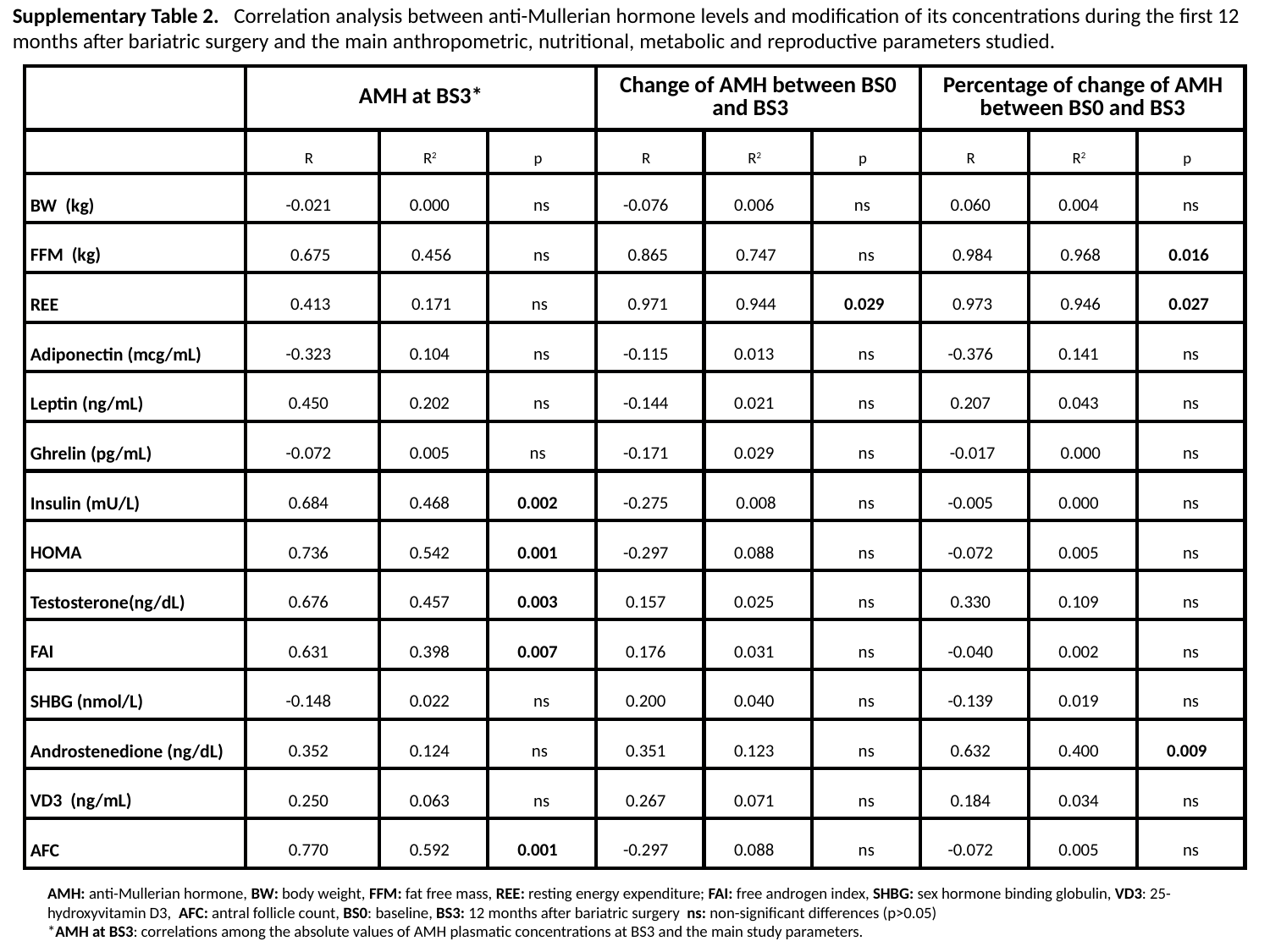

Supplementary Table 2.   Correlation analysis between anti-Mullerian hormone levels and modification of its concentrations during the first 12 months after bariatric surgery and the main anthropometric, nutritional, metabolic and reproductive parameters studied.
| | AMH at BS3\* | | | Change of AMH between BS0 and BS3 | | | Percentage of change of AMH between BS0 and BS3 | | |
| --- | --- | --- | --- | --- | --- | --- | --- | --- | --- |
| | R | R2 | p | R | R2 | p | R | R2 | p |
| BW  (kg) | -0.021 | 0.000 | ns | -0.076 | 0.006 | ns | 0.060 | 0.004 | ns |
| FFM  (kg) | 0.675 | 0.456 | ns | 0.865 | 0.747 | ns | 0.984 | 0.968 | 0.016 |
| REE | 0.413 | 0.171 | ns | 0.971 | 0.944 | 0.029 | 0.973 | 0.946 | 0.027 |
| Adiponectin (mcg/mL) | -0.323 | 0.104 | ns | -0.115 | 0.013 | ns | -0.376 | 0.141 | ns |
| Leptin (ng/mL) | 0.450 | 0.202 | ns | -0.144 | 0.021 | ns | 0.207 | 0.043 | ns |
| Ghrelin (pg/mL) | -0.072 | 0.005 | ns | -0.171 | 0.029 | ns | -0.017 | 0.000 | ns |
| Insulin (mU/L) | 0.684 | 0.468 | 0.002 | -0.275 | 0.008 | ns | -0.005 | 0.000 | ns |
| HOMA | 0.736 | 0.542 | 0.001 | -0.297 | 0.088 | ns | -0.072 | 0.005 | ns |
| Testosterone(ng/dL) | 0.676 | 0.457 | 0.003 | 0.157 | 0.025 | ns | 0.330 | 0.109 | ns |
| FAI | 0.631 | 0.398 | 0.007 | 0.176 | 0.031 | ns | -0.040 | 0.002 | ns |
| SHBG (nmol/L) | -0.148 | 0.022 | ns | 0.200 | 0.040 | ns | -0.139 | 0.019 | ns |
| Androstenedione (ng/dL) | 0.352 | 0.124 | ns | 0.351 | 0.123 | ns | 0.632 | 0.400 | 0.009 |
| VD3  (ng/mL) | 0.250 | 0.063 | ns | 0.267 | 0.071 | ns | 0.184 | 0.034 | ns |
| AFC | 0.770 | 0.592 | 0.001 | -0.297 | 0.088 | ns | -0.072 | 0.005 | ns |
AMH: anti-Mullerian hormone, BW: body weight, FFM: fat free mass, REE: resting energy expenditure; FAI: free androgen index, SHBG: sex hormone binding globulin, VD3: 25-hydroxyvitamin D3,  AFC: antral follicle count, BS0: baseline, BS3: 12 months after bariatric surgery  ns: non-significant differences (p>0.05)
*AMH at BS3: correlations among the absolute values of AMH plasmatic concentrations at BS3 and the main study parameters.
